# High-brightness betatron emission from the interaction of a sub picosecond laser pulse with pre-ionized low-density polymer foam for ICF research

**DOI:** 10.1038/s41598-024-65490-7

**Published:** 2024-06-26

**Authors:** Mikhail Gyrdymov, Jakub Cikhardt, Parysatis Tavana, Nataliya G. Borisenko, Sergey Yu. Gus´kov, Rafael A. Yakhin, Galina A. Vegunova, Wenqing Wei, Jieru Ren, Yongtao Zhao, Dieter H. H. Hoffmann, Zhigang Deng, Weimin Zhou, Rui Cheng, Jie Yang, Jan Novotny, Xiaofei Shen, Alexander Pukhov, Joachim Jacoby, Christian Spielmann, Viacheslav S. Popov, Mikhail E. Veysman, Nikolay E. Andreev, Olga N. Rosmej

**Affiliations:** 1 Institute for Applied Physics (IAP), Goethe University Frankfurt, Frankfurt am Main, Germany; 2 Faculty of Electrical Engineering, Czech Technical University in Prague, Prague, Czechia; 3 Institute of Optics and Quantum Electronics (IOQ), Friedrich Schiller University Jena, Jena, Germany; 4 P. N. Lebedev Physical Institute (LPI), Russian Academy of Sciences, Moscow, Russia; 5 MOE Key Laboratory for Nonequilibrium Synthesis and Modulation of Condensed Matter, School of Physics, Xi’an Jiaotong University, Xi’an, China; 6 Science and Technology On Plasma Physics Laboratory, Laser Fusion Research Center, China Academy of Engineering Physics, Mianyang, China; 7 Institute of Modern Physics, Chinese Academy of Sciences, Lanzhou, China; 8 Center for Applied Physics and Technology, HEDPS, and SKLNPT, School of Physics, Peking University, Beijing, China; 9 Institut für Theoretische Physik I, Heinrich-Heine-Universität Düsseldorf, Düsseldorf, Germany; 10 Joint Institute for High Temperatures, Russian Academy of Sciences, Moscow, Russia; 11 Moscow Institute of Physics and Technology (State University), Dolgoprudny, Russia; 12 GSI Helmholtzzentrum für Schwerionenforschung, Darmstadt, Germany; 13 Helmholtz Forschungsakademie Hessen für FAIR, Frankfurt am Main, Germany

**Keywords:** Physics, Plasma physics, Laser-produced plasmas, Plasma-based accelerators, Engineering

## Abstract

Direct laser acceleration (DLA) of electrons in plasmas of near-critical density (NCD) is a very advancing platform for high-energy PW-class lasers of moderate relativistic intensity supporting Inertial Confinement Fusion research. Experiments conducted at the PHELIX sub-PW Nd:glass laser demonstrated application-promising characteristics of DLA-based radiation and particle sources, such as ultra-high number, high directionality and high conversion efficiency. In this context, the bright synchrotron-like (betatron) radiation of DLA electrons, which arises from the interaction of a sub-ps PHELIX laser pulse with an intensity of 10^19^ W/cm^2^ with pre-ionized low-density polymer foam, was studied. The experimental results show that the betatron radiation produced by DLA electrons in NCD plasma is well directed with a half-angle of 100–200 mrad, yielding (3.4 ± 0.4)·10^10^ photons/keV/sr at 10 keV photon energy. The experimental photon fluence and the brilliance agree well with the particle-in-cell simulations. These results pave the way for innovative applications of the DLA regime using low-density pre-ionized foams in high energy density research.

Optimizing the laser energy coupling into relativistic electrons to achieve the highest possible conversion efficiency when generating laser-driven sources of MeV particles (electrons, protons, neutrons) and radiation is a challenging task for many laser facilities supporting Inertial Confinement Fusion (ICF) research^1,2^. Accelerated electrons can be used to generate X-rays through several processes like betatron emission, electron-driven bremsstrahlung radiation and inverse Compton scattering^3–12^. Betatron emission generated by electrons during laser-plasma acceleration is characterized by a short pulse duration, small source size, broadband X-ray spectrum, and a small divergence. It is confirmed to be promising for X-ray radiography and X-ray phase contrast high-resolution imaging in investigations of ultra-fast processes in High Energy Density Science (HEDS)^13–15^.

Depending on laser and plasma parameters, betatron radiation can be generated in the process of the laser wakefield acceleration (LWFA)^16–18^, self-modulated LWFA (SM-LWFA)^19–21^, direct laser acceleration (DLA)^22,23^ or a hybrid regime^7,24^.

The LWFA works best in tenuous, underdense plasmas and ultra-short laser pulses, shorter than the plasma wave period. The most prominent case of LWFA is the so-called bubble regime^17^. It allows reaching GeV electron energies with total beam charge of ≥ 100 pC^25,26^. Plasma electrons trapped in the cavity experience both longitudinal and radial focusing fields and therefore oscillate transversely as they are accelerated forward. These so-called betatron oscillations cause the electrons to emit synchrotron-like radiation confined to a narrow cone (10–50 mrad) in the forward direction. Due to relative low electron beam charge, total number of X-ray photons reaches ~ 10^9^^26–28^.

Self-modulated LWFA works at higher plasma densities (still much lower than the critical one) and longer laser pulse duration (longer than the plasma wave period). Here, a high-intensity (> 10^19^ W/cm^2^) picosecond laser pulse propagates through an underdense plasma and generates plasma waves via the resonance self-modulation instability (RSMI)^19,20^ or Raman Forward Scattering (RFS)^29^. Betatron-like oscillations of the trapped and accelerated off-axis electrons causing them to radiate X-ray photons in the forward direction with a higher X-ray photon number due to higher number of accelerated electrons compared to the LWFA^4,5^.

A SM-LWFA regime builds a platform for generation of broad band X-ray sources at large kJ-class picosecond laser facilitates in operation such as the advanced radiographic capability (ARC) at the National Ignition Facility (NIF), the Petawatt Aquitaine Laser (PETAL) of the Laser Megajoule (LMJ) in France, OMEGA EP etc.^30^. In experiments performed at the OMEGA EP and the Titan lasers, which support this program, betatron X-ray emission has been produced by irradiation of super-sonic gas-jets with ps laser pulses of moderate relativistic intensity^4,5,7,8^. The bright betatron radiation with > 10^10^ photons/keV/sr at X-ray energies > 15 keV was reported. At the same time, the nature of SM-LWFA based on instabilities, leads to a rather poor reproducibility of results^31^. It has also been shown that SM-LWFA is the main mechanism for accelerating electrons up to energies of ~ 40 MeV, while for higher energies direct laser acceleration (DLA) dominates^24^. The major role of DLA in interaction of the Vulcan ultra-intense laser pulses with a normalized vector potential *a*_0_ > 10 with high density gas-target was also discussed in^32^.

Application of the SM-LWFA regime on the ARC faces difficulties caused by an imperfect focal spot of several tens of micrometers, in which most of the laser energy is sub-relativistic. This makes the efficient generation of relativistic electrons in laser-plasma interaction a major challenge. Williams reports on the ARC performance improvements at near-relativistic intensity using compound parabolic concentrator (CPC) cone targets^33^. This concept suggests that the large fraction of energy contained in the wings of the ARC-beam (∼80%) could be repointed towards the central beam spot. Using this approach, more than tripled increase in average electron energy was found compared to flat targets^33,34^. This is due to a combination of the long scale length plasma confined by the cone tip and the enhanced acceleration mechanism driven by laser turbulence that develops from reflections from the cone walls^33^. On the other hand, to control the SM-LWFA, a near-Gaussian single-mode laser spot is crucial^31,35^. This makes CPC hardly compatible with gas-targets.

Recently, direct laser acceleration of electrons in plasma of near-critical density, where the electron density is up to two orders of magnitude higher than in gas jets, has become increasingly attractive^9,22,23,36,37^. In the case of DLA, the pulse is sufficiently long and intense to create an ion plasma channel with strong radial fields. The radial inhomogeneity of the electron density due to the ponderomotive expulsion of background plasma electrons from the channel creates a radial electrostatic field, and at the same time, the current of accelerated electrons generates an azimuthal magnetic field^22,23,38^.

A relativistic electron trapped in the channel experiences transverse betatron oscillations and gains energy efficiently from the laser pulse when the frequency of the betatron oscillations becomes resonant with the Doppler shifted laser frequency^22,23^. Depending on the plasma density and laser intensity, DLA, SM-LWFA, stochastic heating^39^ or a combination of these mechanisms can be realized. Different from LWFA, the DLA does not generate electrons at very high energies, rather, it produces ample amounts of electrons with Maxwellian-like distribution carrying mega-ampere currents. The effective temperature of these electrons can reach several tens of MeV at moderate relativistic intensities.

Generation of the ultra-bright DLA-based secondary sources of MeV particles and radiation was successfully demonstrated at the sub-PW Nd:glass PHELIX laser facility in Darmstadt Germany. Interaction of the sub-ps pulse of ~ 10^19^ W/cm^2^ intensity^40^ with pre-ionized low-density polymer foams^41^ resulted into an increase in the effective electron temperature, electron energy and beam charge by 10–20 times compared to shots on conventional foil at the same laser parameters^9,37^. The resulting mega-ampere current beam of DLA electrons penetrates a high-Z converter attached to the foam on the back side and generates directed gamma rays with an effective temperature of 10–15 MeV and photon energies exceeding 50 MeV, as observed in the detection of short-living isotopes Au192 and Ta176^9,42^ and high-yield neutrons^11^.

With 40% conversion efficiency of the focused laser energy into electrons (> 1.5 MeV) and ~ 2% efficiency of conversion into gamma rays in the giant dipole resonance region (> 8 MeV) a breakthrough in the efficient production of secondary sources using the DLA regime was demonstrated^9,11,42,43^.

In this work, we experimentally explore betatron emission by irradiation of a pre-ionized low-density polymer foam with sub-ps laser pulse of moderate relativistic intensity and demonstrate the feasibility of a robust high-brightness DLA-based betatron source for applications in HED research.

## Experimental set-up

The experiment was performed at the Petawatt High Energy Laser for Ion eXperiments (PHELIX) at the Helmholtzzentrum GSI Darmstadt^40^. A relativistic laser pulse of 0.75 ± 0.25 ps duration and ~ 10^19^ W/cm^2^ intensity was sent to the foam layer pre-ionized by an additional ns pulse. The estimated focal spot size was 13.5 ± 0.5 µm, the total laser energy after compressor was 52 ± 2 J and the energy concentrated in the FWHM of the focal spot was 15 ± 1 J.

**Figure 1:**
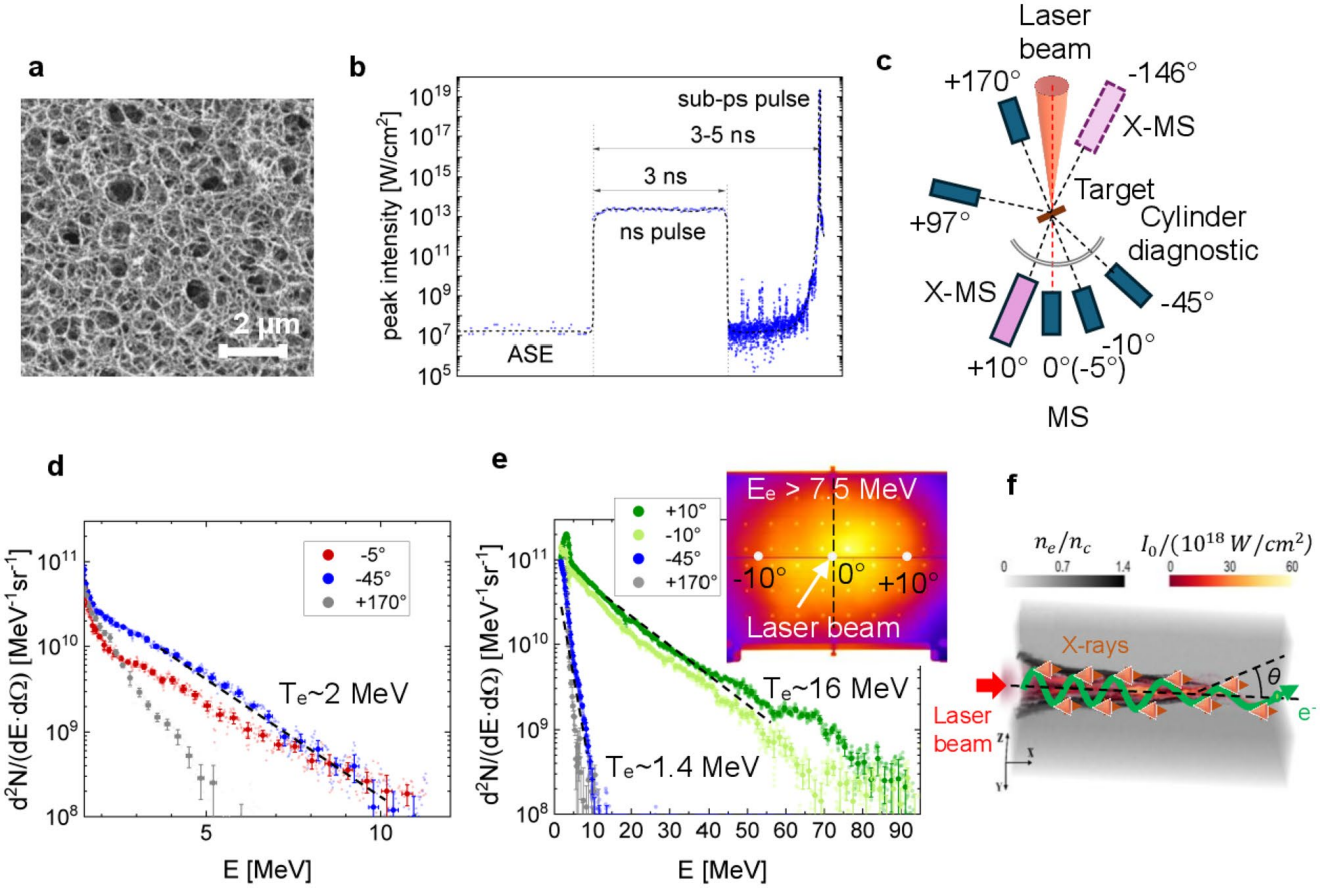
(**a**) Low-density polymer foam with stochastic 3D structure^41^; (**b**) nanosecond pulse to convert the foam into a plasma, followed by a relativistic sub-ps pulse to accelerate the electrons; (**c**) experimental setup with magnetic spectrometers placed around the foam target; (**d**) spectra of the ponderomotive electrons in the case of a high contrast shot on the foam without ns-pulse; (**e**) measured energy and angular distribution of the DLA electrons in the case of pre-ionized foams; (**f**) generation of the synchrotron radiation in the relativistic plasma channel during the betatron oscillations of the DLA electrons.

The sub-microstructure of low-density foam and the profile of the ns pulse forerunning the relativistic PHELIX pulse are shown in Fig. 1a,b correspondently. The foam structure shown in Fig. 1a builds a stochastic 3D micro-network, which consists of ~ 40 nm thick fibers (thicker than the laser skin layer) of 1 g/cm^3^ density and pores of a sub-µm size (more details can be found in ^41^). In the case of fully ionized CHO-plasma, 2 mg/cm^3^ mean density corresponds to 0.64·10^21^ cm^−3^ electron density or 0.64*n*_cr_, where *n*_cr_ = 10^21^ cm^−3^ is a critical electron density for 1 µm laser wavelength. The ns pulse, which was used to ionize foam and convert it into NCD plasma, had a 10^13–^10^14^ W/cm^2^ intensity and 1.5–3 ns duration, depending on the foam thickness and density. It was focused on the target using the same off-axis parabolic mirror with an f/5 number as a relativistic pulse. The generation of a sub-mm long NCD plasma requires focusing optics with a Rayleigh length of hundreds of micrometers, which was the case with PHELIX due to the 150 cm long off-axis parabolic mirror. This ensures a relatively constant ns laser pulse intensity over the entire layer thickness. A delay of 3–5 ns between the ns pulses and the sub-ps pulses was chosen long enough to ionize and homogenize the foam structure.

Since the foam layer was surrounded by a copper disk (washer), measuring the electron density profile at the moment of interaction of the relativistic pulse was difficult. To compensate for this drawback, two-dimensional hydrodynamic (2D HD) modeling of the interaction of the ns pulse with a structured matter was carried out for the foam and ns pulse parameters used in the experiment. An example of simulated plasma density and temperature profiles along the laser axis after exposure to a ns pulse is shown in Fig. 4, “Methods”.

After the action of the ns pulse, the rising edge of the incoming sub-ps laser pulse generates an ion channel where a certain number of electrons is ponderomotively expelled from the Gaussian-like laser beam. Subsequently, in the region with reduced electron density, the remaining electrons undergo transverse betatron oscillations in the quasi-static fields and emit synchrotron-like radiation^10,44^. See also 3D PIC simulations in “Methods” in this work.

For confirmation of the DLA process, where strongly directed super-ponderomotive electrons are expected, measurements were conducted using magnetic spectrometers (MS) placed at different angles to the laser axis. In Fig. 1c, the experimental setup is schematically depicted, employing simultaneously 3–5 MS with ~ 1 T static field and one modified magnetic spectrometer X-MS to measure and to characterize relativistic electrons and X-rays produced in interaction of the sub-ps PHELIX-pulse with plasma. A stack of three stainless half-cylinders charged with imaging plates (IP) was used to measure angular distribution of electrons with energies > 3.5 MeV and > 7.5 MeV. A horizontal 4 mm wide slit centered at the laser pulse height allowed for electron propagation to the magnetic spectrometers placed behind the semi-cylinder-stack^9^. Further details on the electron and keV X-ray diagnostics can be found in “Methods” and “Supplementary Information”.

## Results and discussion

### Measurements of DLA electrons

In Fig. 1d, electron spectra are shown for a 450 ± 50 µm thick 2 mg/cm^3^ CHO-foam, irradiated with a 0.75 ± 0.25 ps pulse of ~ 10^19^ W/cm^2^ intensity and high nanosecond laser contrast of 10^−11^. The resulted spectra show ponderomotive electrons with effective temperature of ~ 2 MeV accelerated in all directions. Similar spectra were observed in shots onto metallic foils.

In Fig. 1e, a ns pulse of a 10^13^ W/cm^2^ intensity and of 3 ± 0.5 ns duration was used to pre-ionize a foam layer. After ns delay, the sub-ps relativistic pulse interacted with the produced plasma. Relativistic electrons with an effective temperature of up to 16 MeV were detected, directed along the laser axis with a half divergence angle of 13 ± 1.5° (inset in Fig. 1e). Electron spectra measured at − 45° to laser axis and in the backward direction show only ponderomotive temperature and much lower electron charge. These results demonstrate the importance of the pre-ionization of polymer foams by a ns pulse for the generation of a homogeneous plasma target.

 Figure 1f shows schematically the generation of synchrotron radiation in the relativistic plasma channel during the betatron oscillations of the DLA electrons.

### Measurement of the betatron radiation

In the interaction of relativistic laser pulse with pre-ionized foam, not only the betatron radiation but also other types of radiation can be produced. The use of a copper ring-like disk (washer) as a holder for the foam leads to bremsstrahlung caused by ponderomotive electrons propagating across the laser axis and interacting with copper. In addition, isotropic self-radiation of the CHO-plasma heated up to 1–2 keV by the relativistic pulse can contribute to measurements at photon energies below 10 keV. To exclude self-radiation of CHO-plasma, we discuss results of measurements made for photon energies above 10 keV.

To separate the betatron radiation from bremsstrahlung, a spatial resolution of the multiple X-ray sources is required. To address this issue, a modified magnetic spectrometer (X-MS) was constructed to provide 1D resolution of the sources and to enable separate observation of the betatron radiation from the NCD plasma and the bremsstrahlung generated in the Cu-washer. The use of a well-shielded X-MS with two magnets, which deflected electrons with energies up to 220 MeV and protons with energies up to 70 MeV, enabled “clean” measurements of the betatron signal. For more details, see “Methods” and “Supplementary Information”.

**Figure 2:**
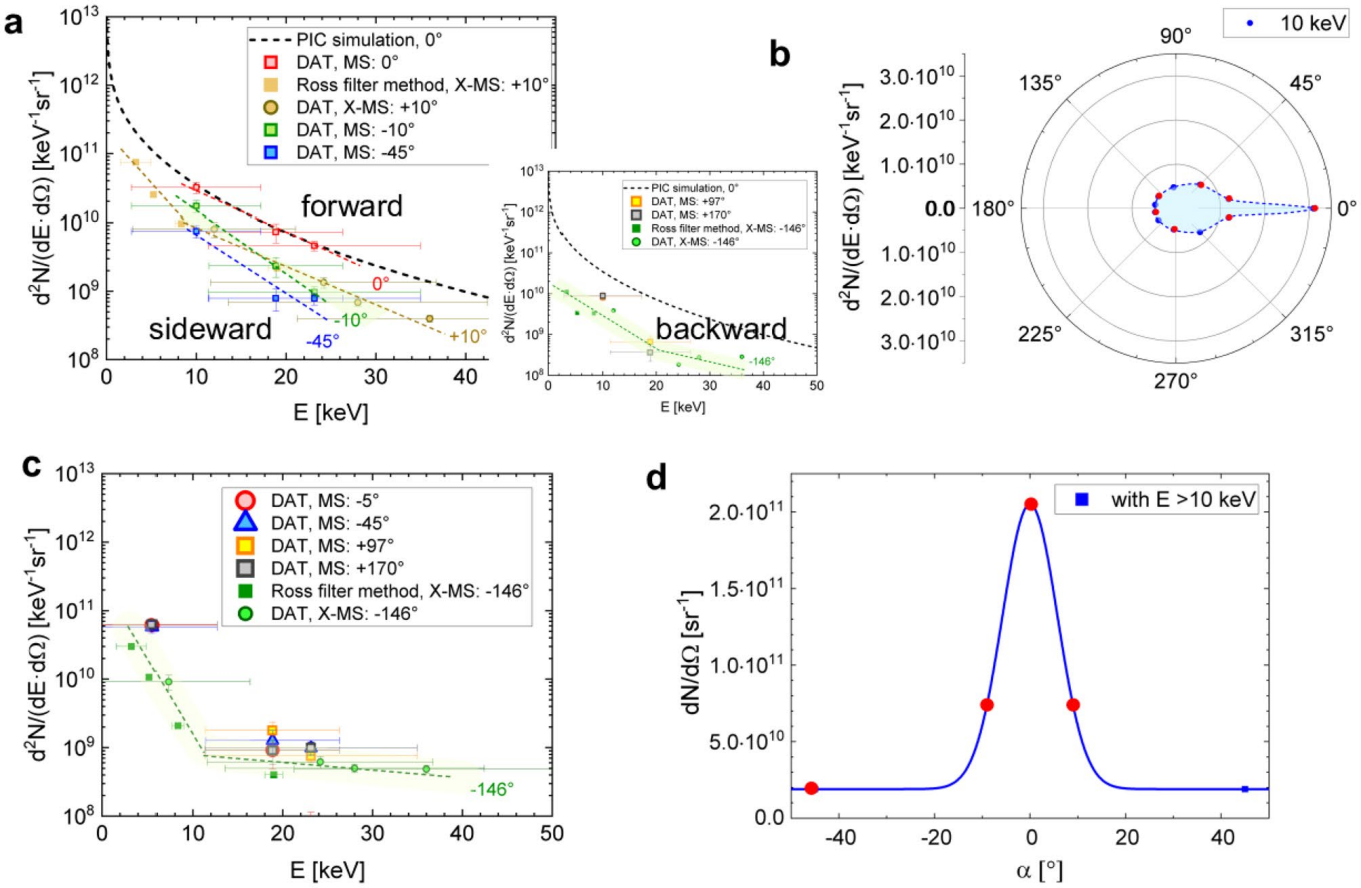
Angular dependent X-ray spectra measured by means of filter methods (laser axis at 0°): (**a**) forward (0°, ± 10°), sideward (− 45°) and backward (− 146°, + 97°, + 170°) radiation in case of pre-ionized foam; black-dashed curve is the result of 3D PIC simulations^10^; (**b**) reconstructed angular distribution of 10 keV photons using MS (− 45°, − 10°, 0°, + 97° and + 170°) and X-MS (− 146°, + 10°) from two subsequent shots; (**c**) X-rays from the interaction of a relativistic pulse with foam without pre-ionization by the ns pulse; (**d**) number of photons/sr with an energy above 10 keV as a function of the angle to the laser axis. Here, the red dots indicate the positions of three MS (− 45°, − 10°, 0°) and one X-MS (+ 10°) in the shot corresponding to the data in Fig. 2a for forward and sideward radiation.

The filter pair method was used to measure the number of X-ray photons/keV/sr at different angles to the laser axis. The X-ray data shown in Fig. 2a were obtained in the case of a 450 ± 50 µm thin foam that was pre-ionized by a ~ 10^13^ W/cm^2^ ns pulse and then irradiated with relativistic pulses of 10^19^ W/cm^2^. The energy errors correspond to the energy windows of the filter pairs, defined as ≥ 50% of the maximum value for the product of the IP transmission difference and the IP sensitivity (more details in the Supplementary Information). The IP stack was simultaneously used for the detection and filtering of X-rays. In the X-MS case, the first IP registered X-rays in the photon energy range between 4 and 19 keV after passing the Ross filter set. The corresponding data points in Fig. 2a,c are denoted as Ross filter method X-MS. X-ray signals filtered by the first, second and third IP of the same stack (DAT X-MS and DAT MS in Fig. 2 a,c) belong to the photon energy range between 12 and 36 keV.

The spectrum along the laser axis (DAT MS 0°) evaluated using the Differential Averaged Transmission (DAT) method^8^, shows a good agreement in the number of photons/keV/sr with 3D PIC (Particle-in-Cell) simulations performed for PHELIX parameters (black dashed line)^10,44^.

The photon number drops with an increasing angle to the laser axis. Already at + 10°, the number of photons measured by means of X-MS using a set of Ross filters (see “Methods") drops up to 4 times at ~ 10 keV photon energy compared to 0°. This result is confirmed by the DAT-method using MS placed at -10° to laser axis. The side (MS at − 45°) and backward (MS at + 97°, + 170° and X-MS at − 146°) radiation exhibit isotropic features and are mainly caused by keV bremsstrahlung from the washer. In Fig. 2b, the angular distribution of ~ 10 keV photons is shown, with red dots depicting the positions of the MS and X-MS spectrometers used for measurements. The directional component corresponds to the betatron radiation generated by DLA electrons in the relativistic plasma channel. The divergence of the X-rays in the directed part is shown in Fig. 2d for photon energies > 10 keV and is summarized in Table 1. In contrast to shots on pre-ionized foam, no directional radiation was observed in shots on foams without the ns pulse (Fig. 2c).

**Table 1:** Characteristics of DLA electrons and the produced betatron radiation.

Shot	Foam target/ thickness (µm) density (mg/cm^3^)	ns pulse: intensity (W/cm^2^)/length (ns)/delay [ns]	Intensity of sub-ps pulse (W/cm^2^)	Directed electrons		Betatron radiation	
				Q_e_ (nC) of DLA electrons with E_e_ > 10 MeV	T_e_ (MeV)	α_1/2 FWHM_ (°)	N_ph_ (× 10^10^ photons/keV/sr) at 10 keV (0° to laser axis)
#12 P21-05-1	CHO/500/2	1·10^13^/3/5	~ 10^19^	30 ± 3	10 ± 1	6.5 ± 1	3.1 ± 0.3
#5 P22-48	CHO/800/2	1·10^14^/3/3	~ 10^19^	39 ± 4	11 ± 1	7.5 ± 1	3.7 ± 0.4
#11 P22-48	CHO/800/2	3·10^13^/3/3	~ 10^19^	37 ± 4	13 ± 1	10 ± 1.5	3.0 ± 0.3
#2 P22-48	CHO/800/2	1·10^13^/3/3	~ 10^19^	34 ± 3	16 ± 2	11 ± 1.5	3.5 ± 0.4
#17 P22-48	CHO/800/2	5·10^13^/1.5/4	~ 10^19^	48 ± 5	16 ± 2	11 ± 1.5	3.0 ± 0.3
Shou (2022)^50^	CNF/40/6	High contrast	~ 10^21^	50 (PIC)	20 (all directions)	> 50° (PIC)	1 (14° to laser axis)

The data in Fig. 2a, obtained at 0° to the laser axis, can also be approximated using Eq. (64) in^45^, averaged over the energies and betatron radii of the emitting DLA electrons in the plasma channel:

1 $$\frac{{d^{2} N}}{{dEd{\Omega }}} = \alpha_{f} \frac{3}{{\pi^{2} }}\frac{{N_{e} }}{2\pi E}\mathop \smallint \limits_{0}^{\infty } {\upgamma }^{2} N_{\beta } f_{e} \left( {E_{e} } \right)dE_{e} \mathop \smallint \limits_{0}^{\infty } dr_{\beta } f_{r} \left( {r_{\beta } } \right)\left( {\frac{E}{{E_{c} }}} \right)^{2} K_{2/3}^{2} \left( {\frac{E}{{E_{c} }}} \right),$$

where ${\alpha }_{f}={e}^{2}/(\hslash c)=1/137$, ${f}_{e}({E}_{e})={T}_{e}^{-1}\text{exp}(-{E}_{e}/{T}_{e})$ is the electrons’ distribution function with electron energy ${E}_{e}$, ${T}_{e}$ is the effective temperature of accelerated electrons, ${N}_{e}$ is their number, $\gamma = {E}_{e}/(m{c}^{2})$, ${f}_{r}({r}_{\beta })=2[\sqrt{\pi }{r}_{\sigma }{]}^{-1}\text{exp}\left[-({r}_{\beta }/{r}_{\sigma }{)}^{2}\right]$ is the distribution function of electrons over the amplitudes of their betatron oscillations ${r}_{\beta }$, ${r}_{\sigma }$ is the characteristic value of ${r}_{\beta }$, ${N}_{\beta }=L/{\lambda }_{\beta }$ is the number of betatron oscillations over ion channel length *L* with the length of betatron oscillations ${\lambda }_{\beta }=2\pi c/{\omega }_{\beta }$ , ${\omega }_{\beta }$ is the betatron frequency of electrons in the ion channel, and ${E}_{c}=\hslash {\omega }_{c}=3{a}_{\beta }{\upgamma }^{2}\hslash {\omega }_{\beta }$ with ${a}_{\beta }=\gamma {\omega }_{\beta }{r}_{\beta }/c$ is the critical energy. In the case of complete evacuation of electrons from the channel, the betatron frequency determined by the electrostatic focusing force equals to ${\omega }_{\beta }={\omega }_{p}/\sqrt{2\gamma }$, where ${\omega }_{p}={(4\pi {e}^{2}{n}_{e}/m)}^{1/2}$ is the electron plasma frequency determined by the unperturbed plasma density.

Good agreement of the photon energy spectrum (1) with the results of measurements and PIC modelling shown in Fig. 2a for 0°, was obtained for an effective temperature *T*_*e*_ = 10 MeV, a characteristic value of betatron radius ${r}_{\sigma }$ = 3 µm, ${n}_{e}/{n}_{cr}=0.1-0.2$ and the number emitting electrons ${N}_{e}\cong (1-3)\cdot {10}^{11}$. These parameters are consistent with the measured characteristics of the DLA electrons shown in Table 1.

Depending on the foam parameters (volume density, structure, chemical composition, and thickness) and characteristics of the ns pulse (intensity, duration, ns – sub-ps delay), plasmas with different density profiles can be produced. Two-dimensional hydrodynamic simulations were performed for 2 mg/cm^3^ 450 µm thick foam layer irradiated with a ns pulse of 10^13^ W/cm^2^ intensity and 3 ns pulse duration using NUTCY-F code^46^. The code accounts for the characteristics of the foam structure, laser radiation absorption and the hydrodynamic and thermal energy transfer in partially homogenized plasma^47–49^. The code uses the equation of state of partially homogenized plasma described in^49^, which considers the contribution to pressure from only the homogenized part of matter (for more details see “Methods”).

The resulting plasma profile, created at the time of arrival of the relativistic pulse with 3 ns delay to the ns pulse, consists of a long-scale underdense part, a sharp density up-ramp in front of the shock where the mass density is 2–3 times higher than initial one, and a part with initial foam density unperturbed by the ns pulse (see Fig. 4a,b, “Methods”).

3D PIC simulations previously performed for a linear ramped density profile followed by a density plateau^37^ and currently for the plasma profile shown in Fig. 4b revealed a weak dependence of the properties of the accelerated electrons on the specific shape and length of the underdense part of the plasma target. 3D PIC simulations show that both the strong laser self-focusing and intense DLA start at the density up-ramp around 0.1*n*_cr_ and continue in the NCD plasma, which is thick enough to fully absorb the laser energy. At the same time, the long-scale, underdense plasma does not contribute significantly to the DLA process (further details will be published elsewhere). These results may explain the high stability of the DLA process when using pre-ionized foams^9^.

This regime occurs until the shock wave reaches the back side of the foam. Thereafter, the plasma expands in both directions and the total plasma density decreases rapidly and reaches a subcritical value close to a gas-jet.

In contrast to NCD foams, where the shock region is still transparent for the laser pulse with moderate relativistic intensity, the shock generated in overdense foams can reflect the relativistic laser pulse back. In this case, the DLA takes place only in the pre-plasma expanded to-wards the laser. Experiments show that in order to increase the plasma scale length and to optimize the DLA in overdense foams, the delay between the ns pulse and the relativistic pulse should be longer than in the NCD case so that the shock can propagate deeper in the foam. In Table 1, several PHELIX shots at laser intensity of ~ 10^19^ W/cm^2^ and various parameters of the ns pulse and NCD foam thickness are presented.

Depending on the ns pulse, the charge of directed electrons (> 10 MeV), which emerged from plasma varies from 30 to 50 nC. In all measurements, the total number of directed 10–30 keV photons and the betatron divergence are lower than predicted by 3D PIC simulations: (1–3)·10^10^ vs. 9·10^10^ and 100–200 mrad vs. 300–400 mrad. This is likely explained by the higher laser energy and laser intensity used in simulations^10^ but also by the difference between the plasma profile after exposure to a ns pulse and uniform NCD plasma slab used in^10^.

As already mentioned, the strong laser self-focusing and the DLA start at the electron density up-ramp in front of the shock (Fig. 4b, "Methods") and continue in the NCD region of the density profile. Depending on the ns pulse intensity, the delay between the ns and sub-ps pulses, and foam thickness, the contribution of these two regions to the resulting spectrum can be different. A long density up-ramp and short NCD part can lead to fewer photons but with a narrower angular distribution. Finally, the photon fluence in the experiment and in the simulations agree well (~ 2·10^11^ photons/sr vs. ~ 3·10^11^ photons/sr). The stable number of ~ 3·10^10^ photons/keV/sr at 10 keV photon energy independent of the ns pulse considered in Table 1 proves once again the high robustness of the DLA-based sources.

**Figure 3:**
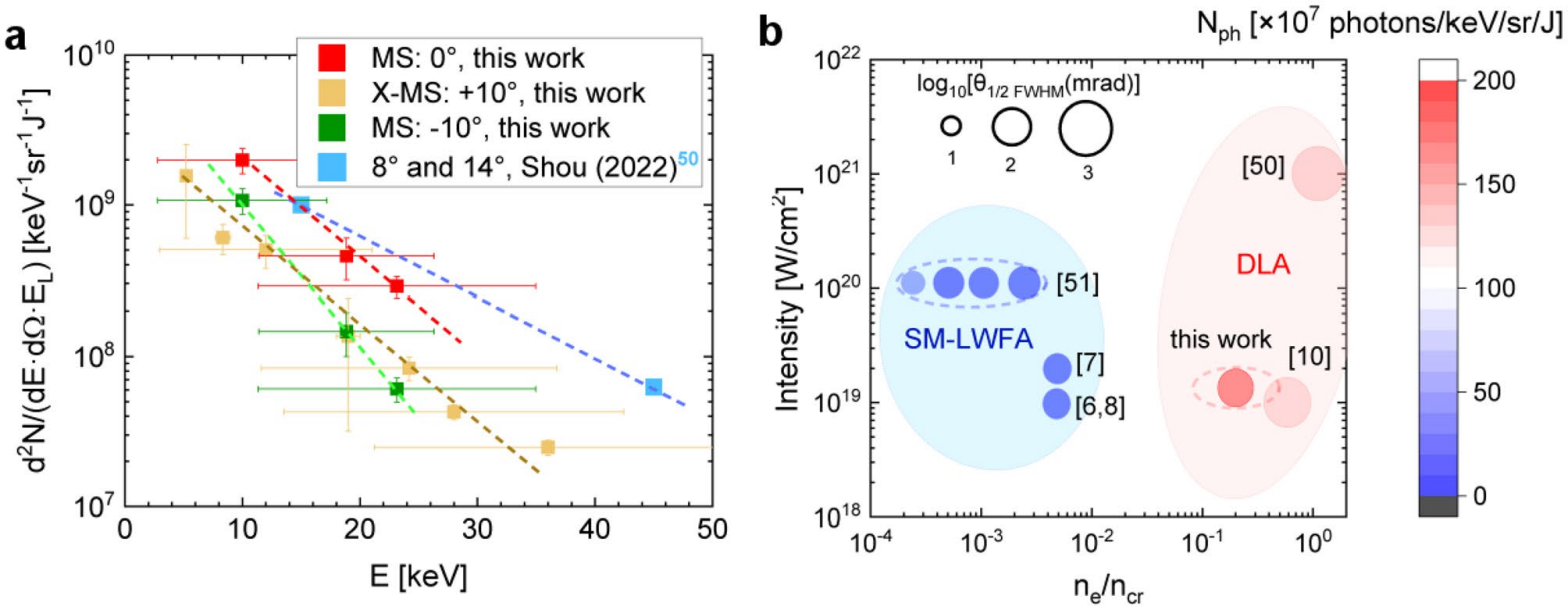
(**a**) Comparison of measured photons/keV/sr normalized to focused laser energy in case of CNF-^50^ and CHO-foams (this work), dotted lines are used to guide the eye; (**b**) number of 10 keV photons per energy bin, solid angle, and laser energy. The diameter of the circles indicates the half-angle (FWHM) of the betatron radiation in the logarithmic scale. The numbered works are as follow: Lemos (2018, 2019)^6,7^, King (2019)^8^, Shen (2021)^10^, Shou (2022)^50^, Ferri (2016)^51^.

In Fig. 3a, betatron spectra acquired at 0° and ± 10° to the laser axis are presented for the PHELIX case (this work) along with the results from Shou et al. obtained at the Center for Relativistic Laser Science (CoReLS) in Korea. There, targets made of carbon nanotube foam (CNF) were irradiated with 20 fs, ~ 10^21^ W/cm^2^ laser pulses and high ns contrast^50^. In both cases, the photon fluence was normalized to the focused energy of each laser (~ 30% from the total energy). Two orders of magnitude higher laser intensity resulted in a much higher total number of X-ray photons compared to PHELIX, but with a betatron divergence of a few steradians predicted by PIC simulations (private communications, Shou et al.). This feature of DLA at ultra-high laser intensities can be explained by the high amplitude of the laser field, which leads to a large transverse moment of DLA electrons and, as a result, a large divergence. This feature of DLA is confirmed by experiments on the Vulcan laser with a normalized amplitude of the laser field *a*_0_ = 10–30^32^. As mentioned above, the betatron radiation at ~ 10^19^ W/cm^2^ laser intensity is well directed and has a half-angle of 100–200 mrad (6.5–11°) (see Table 1).

Summarizing the results on betatron radiation from pre-ionized polymer foams at moderate relativistic intensity and a sub-ps laser pulse, as well as from CNF-foams irradiated with ultra-high laser intensity and a 20 fs laser pulse, one can conclude that in both experiments a comparable number of 10–40 keV photons/sr normalized to laser energy was achieved.

Figure 3b shows numerically predicted and measured number of 10 keV photons per energy bin and solid angle normalized to the focused laser energy^6–8,10,50,51^, which use super-sonic gas-jets and low-density foams as targets irradiated with sub-ps laser pulses (except^50^). As it was mentioned in the introduction, the acceleration regime spans from SM-LWFA and hybrid SM-LWFA–DLA in gas-jets up to DLA in foams. One can conclude that in the SM-LWFA regime, more collimated betatron radiation can be produced but with less number of photons/sr/keV/J compared to numerical predictions^10^ and experiments with foams^50^ and this work.

## Conclusion and outlook

In this work, we studied betatron radiation in a long-scale NCD plasma at moderate relativistic laser intensity and picosecond pulse duration, both typical of currently operating kJ-PW class laser facilities, and demonstrated the feasibility of the high brightness betatron source. The use of low-density foams has opened the door to the practical implementation of DLA in NCD plasma and demonstrated its high robustness.

In the experiment with the Nd:glass PHELIX laser, we measured directed beams of super-ponderomotive electrons and directed X-rays generated in the interaction of a sub-ps 10^19^ W/cm^2^ laser pulse with pre-ionized low-density polymer foams of sub-mm thickness. Electron spectra showed a strong dependence on the direction of observation. The highest charge of the directed part of the electron beam (> 10 MeV) reached 2–3 nC per joule of focused laser energy and the highest effective temperature of 10–16 MeV was measured along the laser axis within the solid angle of 100–200 msr. 3D PIC simulations show near-linear scaling of the DLA-beam charge with laser energy (to be published elsewhere), which is beneficial for the application of foams in kJ and PW class laser systems.

In the case of sub-µm stochastic structure of polymer foam, the importance of applying additional ns pulse to trigger the ionization wave and plasma homogenization process before interaction with relativistic pulse is crucial for DLA. No DLA electrons were observed when shooting foam with high ns laser contrast. Instead, the foam's electron spectra were similar to those obtained by PHELIX pulse interaction with conventional foils (Fig. 1d). This distinguishes it from the nm-thin CNF structure, which provides DLA electrons even at high ns contrast without an additional ns pulse^50,52^, which was also observed in the PHELIX experiments.

The robustness of betatron generation is demonstrated in Table 1, where a stable number of photons/keV/sr at the level of 3·10^10^ for 10 keV photon energy was obtained for different sets of ns pulses. This differs DLA from SM-LWFA based on laser-plasma instabilities.

Another advantage of DLA is that the process happens in high density background plasma. High electron density is responsible for strong transverse focusing force $F_{ \bot }$ acting on the relativistic electrons in the plasma channel. This explains why the photon critical frequency ${\omega }_{c}=\frac{3}{2}{\gamma }^{2}\frac{\left|{F}_{\perp }\right|}{{m}_{e}c}$^53^ differs in the DLA-case by a factor of only 2–5 from LWFA and SM-LWFA, despite significantly lower Lorentz factor γ of accelerated electrons. The critical energy predicted by PIC simulations for PHELIX is 5 keV, while the average γ = 20–30^10^. An additional advantage of the DLA-based betatron source is the small source size of a few micrometers due to the strong self-focusing of the laser pulse during its channel propagation in the NCD plasma^10^.

At the same time, the disadvantage of high electron density (high focusing force) is much higher divergence of synchrotron radiation than in case of LFWA and SM-LWFA accelerators using gas-jets. This starts to be critical at ultra-high laser intensities^32^, what makes Inverse Compton Scattering more advanced as a source of directed X-rays^50^. For PHELIX shots at ~ 10^19^ W/cm^2^, the half opening angle at FWHM of the betatron radiation was estimated from measurements of the X-ray signal at different angles to the laser axis and resulted in 100–200 mrad.

The brilliance of the betatron sources based on the DLA-mechanism in NCD plasma and produced by a picosecond laser pulse can be moderate compared to LWFA, with the record brilliance of 10^23^ photons/s/mm^2^/mrad^2^/(0.1% bandwidth (BW))^27^. The reasons are rather high divergence of the X-ray beam and a picosecond duration of the X-ray flash. In the PHELIX case, estimated peak brilliance of betatron radiation is of ~ 2·10^20^ photons/s/mm^2^/mrad^2^/(0.1% BW) at 10 keV. For this estimation, 5 µm source’s size from 3D PIC simulations was accepted^10^. However, in the case of kJ PW-class lasers, the brilliance of the betatron source is less important compared to the fluence of directed photons used to radiograph an object. This can be explained by the extremely hazard conditions caused by a high flux of particles and photons which interact with target chamber and diagnostic tools and produce a high-level background.

The application of pre-ionized low-density foams to generate a betatron source at the ARC/NIF can be very promising. To meet the 1 ps ARC beam conditions at the PHELIX, the laser beam with 1 ps duration was defocused from 15 µm to 35 µm in diameter and contained ~ 8 J of laser energy with an intensity of 10^18^ W/cm^2^, while the remaining 50 J was below the relativistic limit. Electron beam diagnostics showed an intense beam directed along the laser axis with a temperature of 9 MeV and a charge of 25 nC (> 1.5 MeV). The energy and charge of accelerated electrons decrease sharply as the observation angle increases. Based on these results, the experiment with foams on NIF as part of the Discovery Science program is dedicated to the year 2025.

## Methods

### HD simulations

Two-dimensional hydrodynamic simulations of foam heating by the ns pulse were performed using the NUTCY-F code to reconstruct the density and temperature profiles in the plasma at the time of the relativistic pulse interaction^46^. The NUTCY-F code is one of the versions of the NUTCY Eulerian code^54^ for the modelling of laser–plasma hydrodynamics in an axisymmetric geometry. The absorption coefficient of laser radiation, the equation of state and the thermal conductivity coefficient are calculated in time as a function of the degree of plasma homogenization *H*^47^

2 $$\left( {x,t} \right) \equiv \frac{{m_{h} }}{{m_{0} }} = 1 - \left[ {1 - 2\mathop \smallint \limits_{0}^{t} \frac{{dt^{\prime}}}{{\tau \left( {x,t^{\prime}} \right)}}} \right]^{1/2} .$$

In this expression, ${m}_{h}$ and ${m}_{0}$ are, respectively, the mass of the pore homogenized up to the moment *t* and the total mass of the pore; *τ* is the time of complete homogenization of the individual pore due to diffusion broadening of dense elements of the medium (initially, the pore walls) in the process of ion–ion collisions of plasma flows inside the pores^55^:

3 $$\tau = \frac{{\delta_{0}^{2} }}{{V_{i}^{2} \tau_{ii} }} \approx 2.4 \cdot 10^{ - 11} \frac{{Z^{4} \delta_{0}^{2} \rho }}{{A^{1/2} T^{5/2} }},$$

where *V*_*i*_ is the ion velocity of colliding plasma flows, *τ*_*ii*_ is the time of ion-ion collisions, *T* is the temperature of heated pore’s walls in keV, *ρ* is the average density of porous substance in g/cm^3^, ${\delta }_{0}$ is the average pore’s size in µm.

The absorption coefficient of laser radiation in HD equation of energy is calculated in accordance with the degree of homogenization as the sum of the inverse bremsstrahlung absorption coefficient for the homogenized part of the plasma with a weight factor *H* and the inverse value of geometric transparency length of the non-homogenized part of the plasma with a weight factor $(1-H)$^46,47^. The geometric transparency length is^55^

4 $$L \approx 5 \cdot 10^{ - 4} \cdot \left( {\frac{{\rho_{s} }}{\rho }} \right)^{1 - \alpha } \delta_{0} ,$$

where *ρ*_*s*_ is the density of pore’s wall material; *α* is the fractal parameter, which is equal to 0.8 for micro-size porous substances having, as a rule, a mixed membrane-filament structure.

It is assumed that the non-homogenized part of the plasma does not contribute to pressure and electronic thermal conductivity. The pressure *P*_ph_ in HD equations of motion and energy and thermal diffusion coefficient *χ*_ph_ in HD equation of motion are calculated from the corresponding values of a completely homogenized plasma as *P*_ph_ = *H·P*_h_ and *χ*_ph_ = *H·χ*_*h*_^49^_._

**Figure 4:**
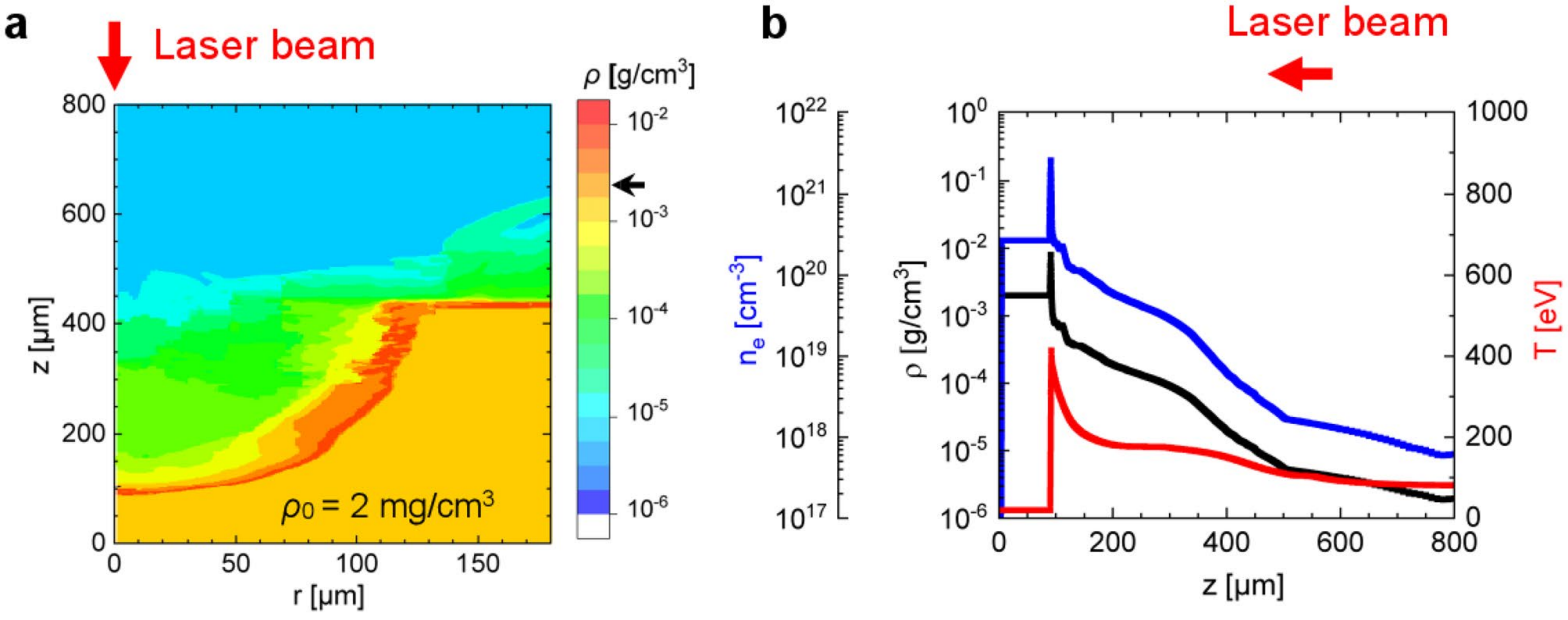
(**a**) Two-dimensional mass density profile 3 ns after interaction of the ns pulse with structured foam with z as the cylindrical axis of symmetry and the direction of laser pulse propagation, while r axis corresponds to radial direction in foam disk (x, r in µm). Foam parameters: 2 mg/cm^3^ mass density, 450 µm thickness and 180 µm radial size. The black arrow on the color bar indicates the initial foam density. (**b**) Profiles of density and temperature generated up to the moment of relativistic pulse interaction with plasma: mass density in g/cm^3^ (black), electron density, cm^−3^ (blue) and electron temperature in eV (red) along z axis.

By varying the ns pulse intensity, which defines the velocity of the ionization wave and the time of structure homogenization, as well as the delay between the nanosecond and relativistic laser pulses, one can create plasmas with different density profiles. Figure 4a,b show results of numerical simulations made for 2 mg/cm^3^ 450 µm thick CHO-foam layer and ns pulse of ~ 10^13^ W/cm^2^ intensity and 3 ns duration used in the experiment. At this laser intensity, the longitudinal ionization wave velocity reaches ~ 100 µm/ns, while in the radial direction it is 2–3 times slower. The homogenization time depends on electron temperature as *T*^-5/2^ and is of 100 ps for 200 eV.

During the propagation of the ns pulse, plasma is partially blown out of the interaction region, which is shown in Fig. 4a, where a blue-green region represents an underdense plasma. Here, the r-axis shows a foam density distribution in the radial direction and the z-axis shows the foam density distribution along the laser pulse axis. The red colored area denotes the shock that propagates within the foam and in the radial direction and has a mass density about twice the original; The yellow colored region represents an area unperturbed by the ns pulse with an initial foam mass density of 2 mg/cm^3^. Figure 4b shows the density and temperature profiles along the z-axis produced up to the time of relativistic pulse interaction with the plasma. All three regions are then completely ionized by the optical field of relativistic laser pulse.

### Electron diagnostics

The angle-dependent energy distribution of relativistic electrons emerging from the plasma was measured using a series of magnetic spectrometers. The permanent magnetic field of ~ 1 T enables the measurement of electrons up to ~ 100 MeV. The energy dispersion was simulated for each MS using a two-dimensional magnetic field distribution measured at the height of the entrance slit. The field distribution between two magnets was very flat with sharp gradients near the spectrometer walls. Thanks to the 300 µm thin entrance slit, an estimated electron energy measurement accuracy of ~ 2% was achieved. The calibration curve for the BASF-MS IP and the FLA-7000 IP scanner from^56^ was used to evaluate the electron energy distribution. The spectrometers were positioned at distance of 400 mm from the target chamber center (TCC) at different angles to the laser axis, as shown in Fig. 1c.

To record the angular distribution of electrons, a stack of three stainless half-cylinders, each 3 mm thick and with a radius of curvature of 300 mm, was used at distance of 300 mm from the TCC. Large area BAS SR- or TR-type imaging plates were placed between the first and second, the second and third half-cylinders to image the angular distribution of electrons with *E* > 3.5 MeV and E > 7.5 MeV, respectively.

A horizontal, 4 mm wide slit centered at the height of the laser pulse allowed electron propagation to the magnetic spectrometers placed behind the cylinder stack. To map the position of the electron beam in space, small holes were drilled in the front panel at 20 mm intervals in vertical and horizontal directions. The result of this diagnostic is shown in the inset of Fig. 1e.

### X-ray diagnostics

**Figure 5:**
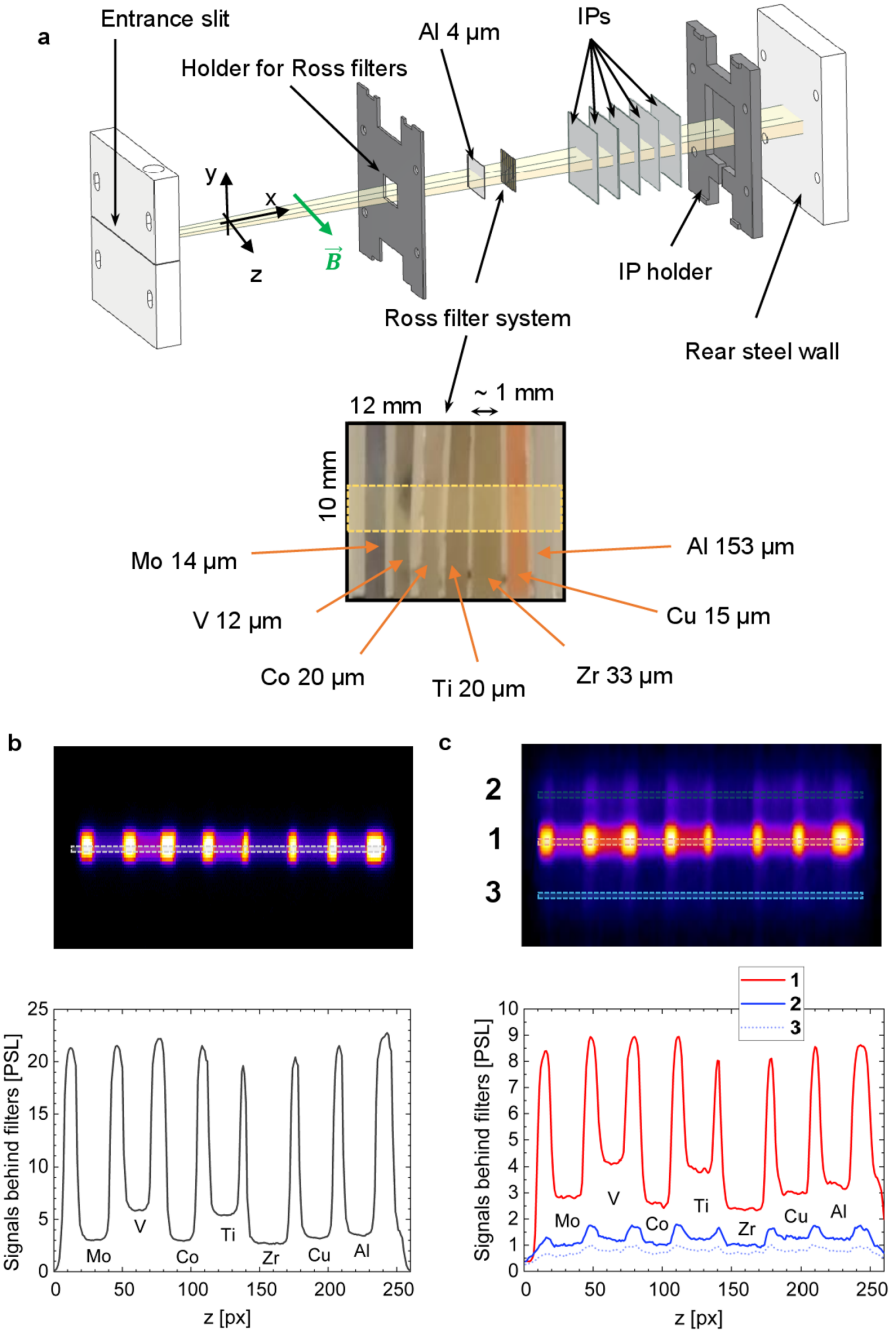
(**a**) Setup of the Ross filters in the modified magnetic spectrometer. (**b**–**c**) X-ray signals after passing Ross filters and registered by X-MS: for Au-foil irradiated by a relativistic laser pulse at high contrast (**b**), for pre-ionized CHO-foam irradiated by a relativistic laser pulse (**c**). Numbers 1, 2, 3 denote betatron (1) and bremsstrahlung (2, 3) signals and their profiles in PSL after passing the Ross filters. (**b**, **c**), 1 pixel corresponds to 50 µm on IP.

MS and X-MS were used to measure X-rays. In both devices, the X-ray signal is projected onto the back wall through an entrance slit, where a series of filters and IPs are placed as detectors. The primary distinction between the two spectrometers is the inclusion of a Ross filter system in the X-MS, as it is shown in Fig. 5a (see also “Supplementary Information” for more technical details).

The modified magnetic spectrometer incorporates several features to measure X-ray radiation from external sources. It utilizes stepped entrance slits to reduce background noise. To avoid input of electrons (up to 220 MeV) and protons (up to 70 MeV) into the X-rays’ signal measured by IPs, permanent magnets were used.

In the rear section of the magnetic spectrometer, a Ross filter system is used (Fig. 5a), followed by a series of successive IPs. Figure 5b,c show two examples of X-ray signals on IP after passing through the Ross filters set and recording with X-MS. Figure 5b displays a 1D image of the self-radiation of keV hot Au-plasma heated by a relativistic PHELIX pulse, while the central part of Fig. 5c (area 1) depicts intense betatron radiation of NCD plasma and rather weak bremsstrahlung from the Cu-washer (areas 2, 3) in the shot onto pre-ionized foam.

The Ross filter and the Differential Average Transmission (DAT) methods^8^ enable the evaluation of X-ray spectra. A correction to the Ross filter method^8^ was used, involving iterative assessments that consider signals outside the energy window of a Ross filter pair. This correction procedure is described in more detail in the “Supplementary Information” to the paper.

### Python simulation

To better understand the geometry of the X-ray source projected through the entrance slit of X-MS onto the detectors (IPs), Python code using geometric optics was used. Signals from various spatially separated sources have only 1D resolution. This means that signals from different sources may overlap in some cases, particularly in the case of a “ring X-ray source” (the washer produces bremsstrahlung) and a central X-ray source (plasma channel) of the target. In this case, it is possible to separate sources by modeling the geometry of the sources. In our case, the effect of bremsstrahlung on the betatron radiation signal is noticeably weaker (Fig. 5c). However, it is possible to exclude the contribution of bremsstrahlung by applying appropriate corrections.

**Figure 6:**
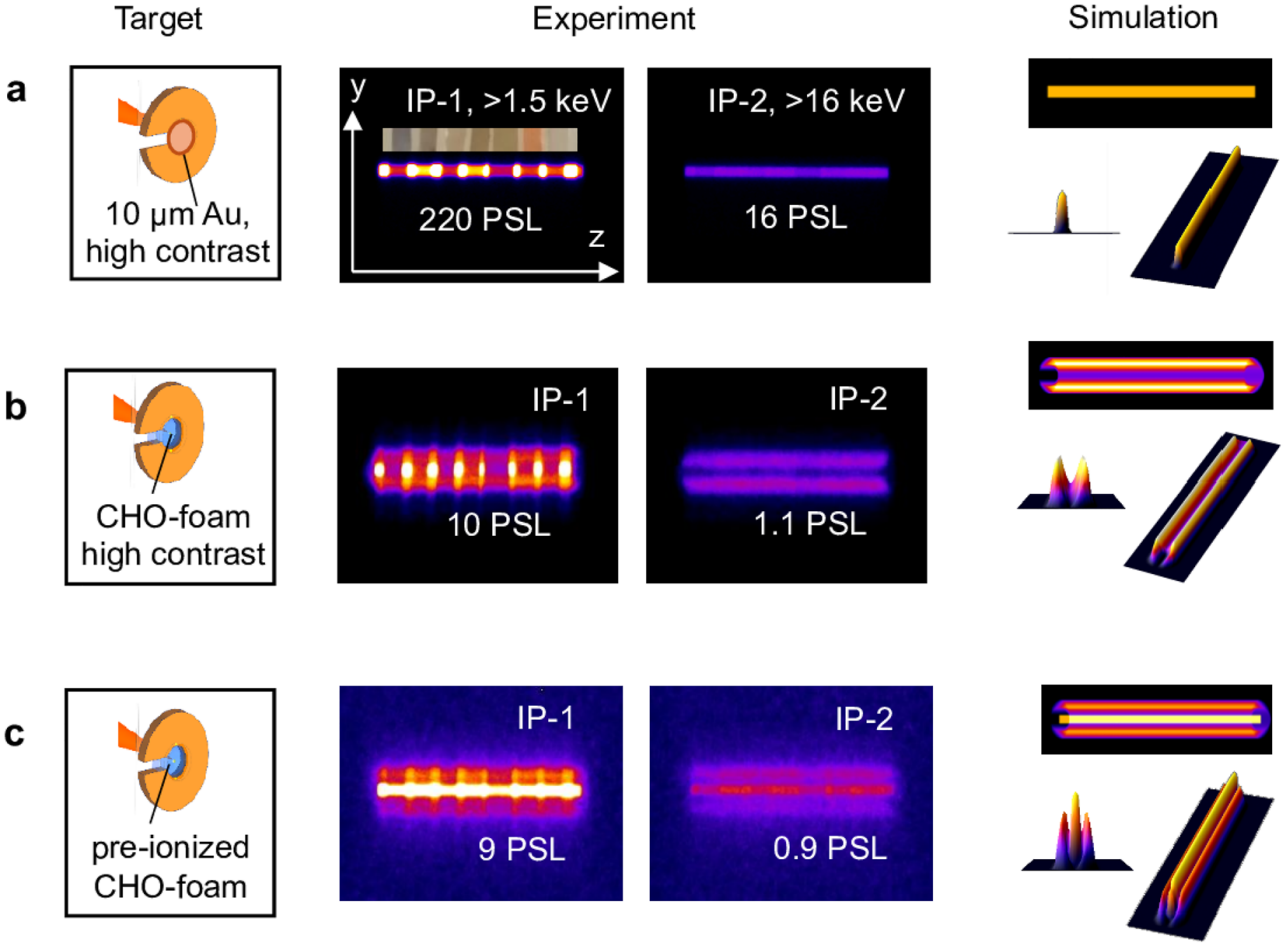
Experimental and simulated 1D images of the X-ray sources on the first and second IPs behind a set of Ross filters in case of: (**a**) High contrast shot at 10^19^ W/cm^2^ laser intensity on Au-foil; (**b**) High contrast shot at 10^19^ W/cm^2^ on CHO-foam; (**c**) Shot at 10^19^ W/cm^2^ on CHO-foam pre-ionized by the ns pulse.

When imaging the X-ray source through the entrance slit in the MS and X-MS, the criterion of geometric optics is fulfilled for X-ray radiation with energies in the range of a few keV with $d\gg \sqrt{L\lambda }$, where *d* is the width of the entrance slit in magnetic spectrometer. *L* is the distance between the center of target and entrance slit and* λ* the wavelength of the X-ray radiation. In our case, *d* ~ 300 µm and *L* ~ 400 mm. For X-ray radiation with *E*_γ_ ≥ 1 keV, the corresponding wavelength is *λ* ≤ 1.24 nm. This condition allows simulating the geometry of the X-ray source and comparing it with experimentally measured signals. Consequently, it is possible to identify the shape or geometry of the source. In Fig. 6a–c, different cases are depicted:

In case a, a 10 µm Au-foil was shot by relativistic pulse at high laser contrast and best focus (~ 15 µm), This resulted in a 1D projection of the point X-ray source on the IP (Fig. 6a, experiment) what is confirmed by the modeling.

In case b, the foam confined in the Cu-washer was irradiated by the relativistic laser pulse at high ns contrast, without pre-ionization by a ns pulse. On the first IP, a strong central signal caused by soft X-rays (> 1.5 keV) was observed, indicating radiation from CHO-plasma. On the second IP (> 16 keV), only lateral signals were registered. The modeling showed that the source, in this case, has a ring-like form and corresponds to a source with a diameter equal to the internal diameter of the Cu-washer. One can conclude that the bremsstrahlung from the Cu-washer holder was registered.

In case c, the foam target was pre-ionized by the ns pulse before the sub-ps relativistic laser pulse arrived. Three signals at low (> 1.5 keV) and higher energies (> 16 keV) were detected in this situation. The central signal corresponds to the betatron radiation from DLA electrons, while at ~ 1.5 keV, plasma self-radiation also contributes. The lateral signals correspond to bremsstrahlung from the Cu-washer holder. The modeling demonstrates a combination of point and ring sources.

### 3D PIC simulations

Simulations of the synchrotron radiation caused by DLA electrons^10^ were performed using the Virtual Laser Plasma Laboratory (VLPL) code^57,58^. The PHELIX laser pulse intensity was approximated using a Gaussian distribution in both temporal and spatial dimensions. The laser focal spot with an elliptical shape was taken from the experiment. The laser pulse energy, confined within the FWHM focal spot of 20 J and a FWHM pulse duration of 700 fs, resulted in a laser intensity of 2.5 $\cdot$ 10^19^ W/cm^2^, corresponding to a_0_ = 4.28.

The simulations considered the ion type and ion fraction based on the chemical composition of triacetate cellulose C_12_H_16_O_8._ The homogeneous NCD plasma of 0.65*n*_c_ was composed of electrons and fully ionized ions of carbon, hydrogen, and oxygen. The simulation box had dimensions of 350λ × 75λ × 75λ and is sampled by 3500 × 150 × 150 cells in *x*, *y*, and *z* directions, where x was the propagation direction of the laser pulse. The numbers of particles per cell in the simulation are four for the electrons and one for the ions of each type (C, H, O). Absorbing boundary conditions are used for particles and fields in each direction. The order of the particle shape function used to describe the NCD plasma is zero. We compared different particle shape functions and found that the difference is negligible. The time step was 0.1 µm along the laser axis (x) and 0.5 µm in the y and z directions. A convergence test was carried out by comparing the physical quantities of interest at different resolutions. It is shown that at the resolution used in the present manuscript, the number of energetic electrons is only about 3% higher than at a finer resolution (${h}_{x}=0.05\lambda , {h}_{y}={h}_{z}=0.3\lambda$).

In VLPL, at each time step, relativistic electrons were assumed to emit photons in the direction of their propagation with a spectrum defined by $S$($\omega /{\omega }_{c}$)^58^, where ${\omega }_{c}$ is a critical frequency. To facilitate the calculation, the critical frequency was determined from the instantaneous acceleration as: ${\omega }_{c}=\frac{3}{2}{\gamma }^{2}\frac{\left|{F}_{\perp }\right|}{{m}_{e}c}$, where ${F}_{\perp }$ is the transverse force felt by electrons at the particular time step. For parameters of the PHELIX experiment it resulted into the critical energy of *E*_c_ = 5 keV. The spectral shape was based on synchrotron (betatron) radiation^59^, with a spectrum determined by the universal function $S\left(x\right)=x{\int }_{x}^{\infty }{K}_{5/3}(\xi )d\xi$ with $x=\omega /{\omega }_{c}$ and ${K}_{5/3}$ is a modified Bessel function of the second kind.

**Figure 7:**
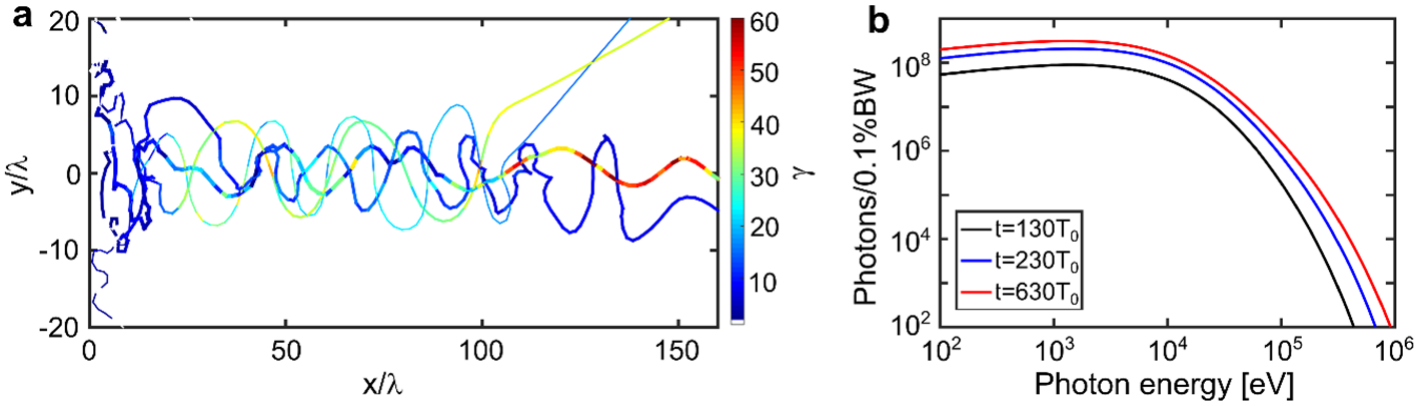
(**a**) Trajectories of four selected electrons with a corresponding relativistic Lorentz factor γ that undergo betatron oscillations; (**b**) Photon energy distribution per 0.1%BW at time *t* = 130*T*_0_ (black), 330*T*_0_ (blue), and 630*T*_0_ (red)^10^.

Figures 7a,b show trajectories of four selected electrons with a corresponding relativistic Lorentz factor γ that undergo betatron oscillations and photon energy distribution per 0.1% BW at time *t* = 130*T*_0_ (black), 330*T*_0_ (blue), and 630*T*_0_ (red). Here the time *t* = 0 corresponds to the peak intensity of the laser pulse on the target front side, for more details on simulations, see in^10^.

Simulations predicted ~ 7·10^11^ photons with energy > 1 keV, more specifically, there are 6·10^11^ photons in the range of 1 keV to 10 keV and 9·10^10^ in the range of 10–30 keV. X-rays are emitted predominantly in the forward direction and most of the photons are concentrated within the angle of ~ 15°. The half opening angle at half maximum (RMS) $\Theta$_y_ (polarization direction) and $\Theta$_z_ at the critical energy are 360 mrad and 270 mrad. Due to the strong self-focusing, the transverse sizes of the radiation source are much smaller than the initial laser focus spot size and approaches ~ 3.5 µm × 4 µm (RMS) along y and z directions. Considering all characteristics of the betatron source, we come up with a peak brilliance of 3.3·10^20^ photons/s/mm^2^/mrad^2^/0.1% BW. More details can be found in^10^.

### Supplementary Information

Supplementary Information.

## Data Availability

The data supporting the plots of this article and other results of this study are available from the corresponding authors on reasonable request.
