# Supplementary material for: High-brightness betatron emission from the interaction of a sub picosecond laser pulse with pre-ionized low-density polymer foam for ICF research
Source: Sci Rep. 2024 Jun 26;14:14785. doi: 10.1038/s41598-024-65490-7 (PMC11208620; doi:10.1038/s41598-024-65490-7)
Supplement: Supplementary file 1 — Supplementary Information. [file 41598_2024_65490_MOESM1_ESM.pdf]

Supplementary Information

Description of X-MS, technical parameters

The modified magnetic spectrometer (Fig. 1S) incorporates several improvements that allow measuring X-ray radiation from laser-target interaction. It uses a set of stepped entrance slits to reduce background noise while also avoiding clipping of information from laterally distributed sources.

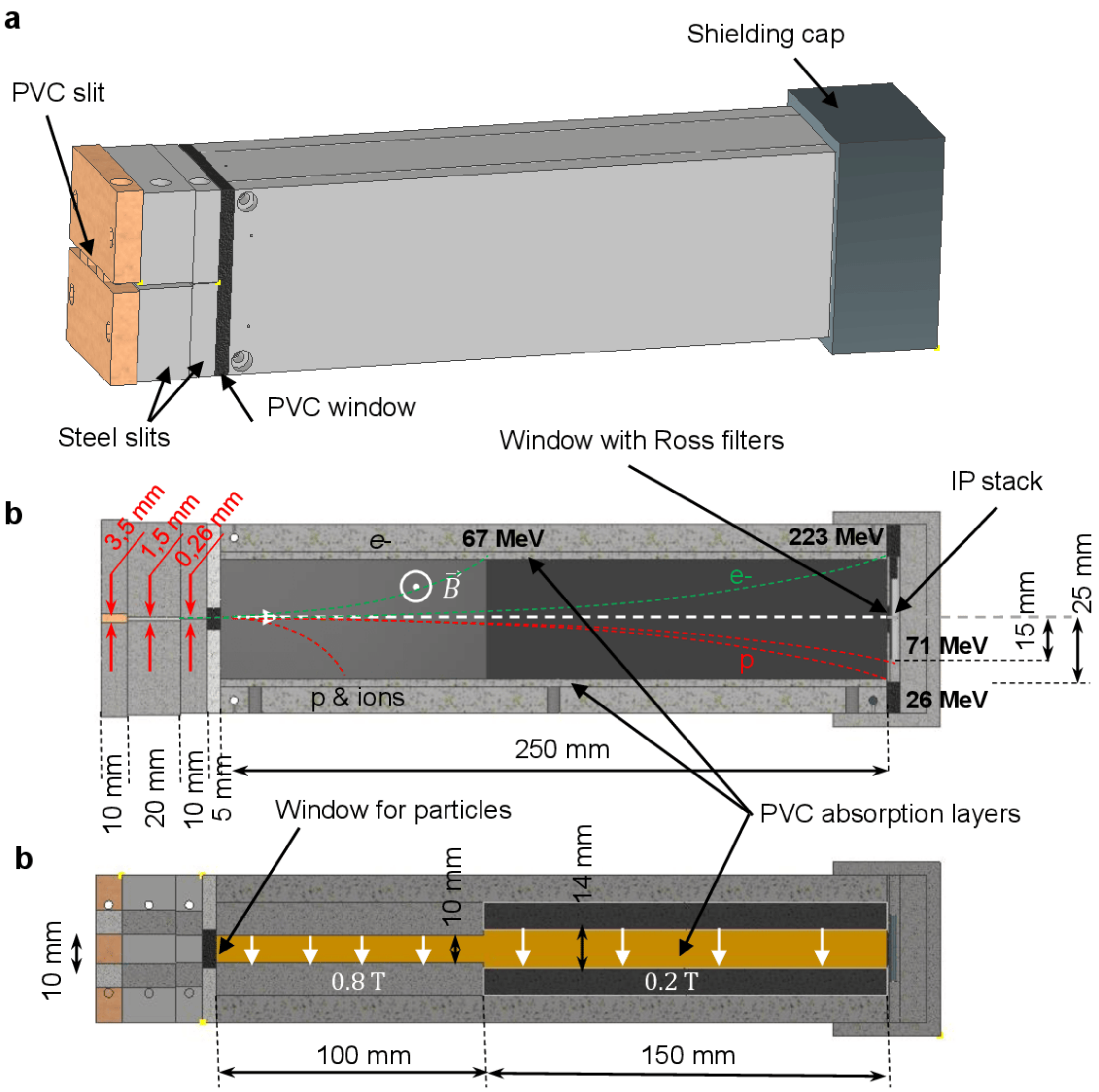

Fig.1S: a. Detailed drawing of the modified magnet spectrometer for X-ray radiation registration (X-MS). **b.** Side view of the X-MS, shown in a central vertical section. **c.** Top view of the X-MS, shown in a central horizontal section.

X-ray signal is registered on the wall of the X-MS opposite to the entrance slit, using a set of Ross filters followed by an IP-stack. Electrons with energies up to 220 MeV and protons with energies up to 70 MeV are deflected to the side by pairs of permanent magnets with strengths of 0.8 T and 0.2 T. Since particles of such high energy have not been observed in experiments, their influence on the registered X-ray signal can be excluded.

Using Ross filter system for spectra evaluation

Table 1S presents the main parameters of the used Ross filters, including material, thickness, and K-edge energy.

Table 1S. Ross filter system in X-MS

Filter 1				Filter 2				<E> [eV]	ΔE [eV]
Material	d_1 [μm]	Δd_1 [μm]	E_1 [eV]	Material	d_2 [μm]	Δd_2 [μm]	E_2 [eV]		
Ti	20.2	0.7	4966	Al	153.9	1.4	1560	3263	1703
V	12.2	0.9	5482	Ti	20.2	0.9	4966	5224	258
Cu	15.2	1.0	8979	Co	20.9	1.7	7710	8345	635
Mo	14.8	0.7	20000	Zr	33.3	1.1	17998	18999	1001

The image of the Ross filter system consisting of seven filters is shown in Fig. 5a, “Methods”. Prior to Ross filters, the radiation undergoes attenuation in a 4 μm aluminum foil, which serves as an overall filter to reduce the intensity of soft X-rays below 0.5 keV (characteristic radiation of CHO-plasma). The transmission of the filters and the transmission difference for the Ross filter pairs are shown in Fig. 2S a and b, respectively.

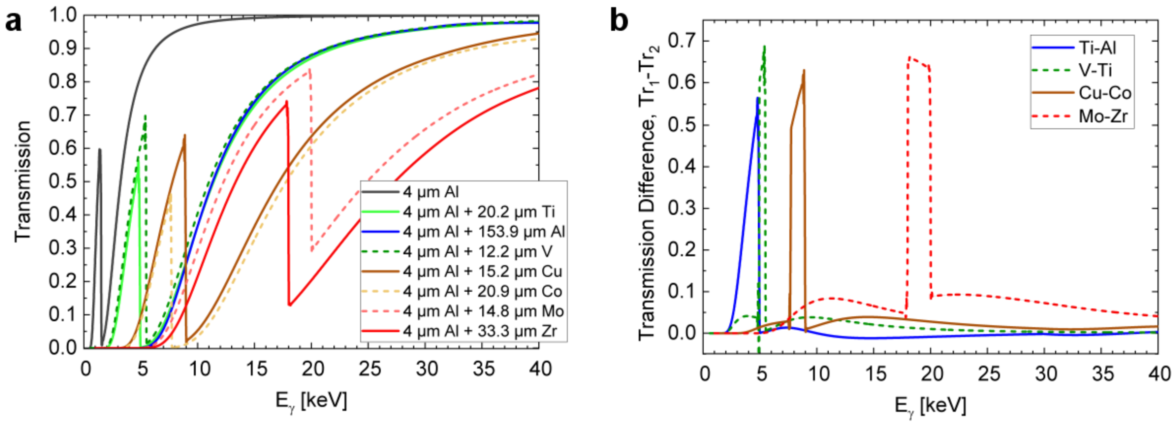

Fig. 2S: a. Transmission of the applied filter. **b.** Transmission difference for Ross filter pairs.

The evaluation procedures begin with the general representation of the signal difference after the Ross filter pair:

$$S_i - S_j = \theta_z \int_0^{+\infty} f_\gamma(E) \cdot \Delta\Omega \cdot \left(Tr_i(E) - Tr_j(E)\right) \cdot S_{IP}(E) \cdot dE, \quad (1)$$

Here, S_i, S_j represents the signal [PSL/px] on the IP after the i-th filter, θ_z is the fading factor of IP signal, $f_\gamma(E)$ is the photon energy distribution function, ΔΩ is the solid angle of observation of the X-ray source on the IP within one pixel ($\Delta\Omega = \left(\frac{l_{1px}}{L_{IP}}\right)^2$, where L_{IP} is the distance between the X-ray source and the IP detector after the filter, and l_{1px} is the size of one pixel on the IP), $Tr_i(E) \cdot S_{IP}(E)$ is the product of the transmission of the i-th filter and the IP sensitivity.

Since $Tr_i(E) - Tr_j(E)$ differs from zero only within the energy window from E_1 to E_2, an approximation can be used for the general expression (1):

$$S_i - S_j \cong \theta_z \int_{E_1}^{E_2} f_\gamma(E) \cdot \Delta\Omega \cdot \left(Tr_i(E) - Tr_j(E)\right) \cdot S_{IP}(E) \cdot dE. \quad (2)$$

Furthermore, assuming that the distribution function f_γ within the energy interval from E_1 to E_2 changes insignificantly, the following approximation holds:

$$f_{\gamma 0} = f_{\gamma 0}\big|_{E_1,E_2} \cong \frac{S_i - S_j}{\theta_z \cdot \Delta\Omega \cdot \int_{E_1}^{E_2} \left(Tr_i(E) - Tr_j(E)\right) \cdot S_{IP}(E) \cdot dE}. \quad (3)$$

Therefore, it is possible to estimate different points in the X-ray spectrum using equation (3). Due to the approximation (3), the error of the method increases. However, the use of the estimation (3) allows for a "zero approximation" of the photon distribution function to be obtained.

Since the transmission difference of the Ross filter pair is non-zero outside the energy window, an error arises in the Ross filter method. To reduce this error, a calculation correction can be applied to the Ross filter method. The values obtained through equation (3) can be regarded as the values of the distribution function in a "zero-approximation". For a more accurate calculation, it is necessary to use the original general equation (1).

$$S_i - S_j = \theta_z \int_0^{+\infty} f_\gamma(E) \cdot \Delta\Omega \cdot \left(Tr_i(E) - Tr_j(E)\right) \cdot S_{IP}(E) \cdot dE, \quad (4)$$

or

$$S_i - S_j = I_1 + I_2 + \theta_z \int_{E_1}^{E_2} f_\gamma(E) \cdot \Delta\Omega \cdot \left(Tr_i(E) - Tr_j(E)\right) \cdot S_{IP}(E) \cdot dE \quad (5)$$

with

$$I_1 = \theta_z \int_0^{E_1} f_\gamma(E) \cdot \Delta\Omega \cdot \left(Tr_i(E) - Tr_j(E)\right) \cdot S_{IP}(E) \cdot dE, \quad (6)$$

$$I_2 = \theta_z \int_{E_2}^{+\infty} f_\gamma(E) \cdot \Delta\Omega \cdot \left(Tr_i(E) - Tr_j(E)\right) \cdot S_{IP}(E) \cdot dE \quad (7)$$

Furthermore, equation (5) is used for an iterative calculation of the desired distribution function f_γ. In the correction terms I_1 and I_2, the "zero-approximation function" $f_{\gamma 0}$ is employed, while in the remaining integral, the function f_γ is taken out of the integral under the assumption that the distribution function f_γ undergoes negligible changes in the energy interval from E_1 to E_2. Consequently, it is possible to compute the "first-approximation function" $f_{\gamma 1}$.

$$f_{\gamma 1} = f_{\gamma 1}\big|_{E_1,E_2} \cong \frac{S_i - S_j - I_{10} - I_{20}}{\theta_z \cdot \Delta\Omega \cdot \int_{E_1}^{E_2} \left(Tr_i(E) - Tr_j(E)\right) \cdot S_{IP}(E) \cdot dE} \quad (8)$$

with

$$I_{10} = \theta_z \cdot \Delta\Omega \int_0^{E_1} f_{\gamma 0}(E) \cdot \left(Tr_i(E) - Tr_j(E)\right) \cdot S_{IP}(E) \cdot dE, \quad (9)$$

$$I_{20} = \theta_z \cdot \Delta\Omega \int_{E_2}^{+\infty} f_{\gamma 0}(E) \cdot \left(Tr_i(E) - Tr_j(E)\right) \cdot S_{IP}(E) \cdot dE. \quad (10)$$

The iterative calculations can be continued for the k-th iteration as follows:

$$f_{\gamma,k} = f_{\gamma,k}\big|_{E_1,E_2} \cong \frac{S_i - S_j - I_{1,k-1} - I_{2,k-1}}{\theta_z \cdot \Delta\Omega \cdot \int_{E_1}^{E_2} \left(Tr_i(E) - Tr_j(E)\right) \cdot S_{IP}(E) \cdot dE} \quad (11)$$

with

$$I_{1,k-1} = \theta_z \cdot \Delta\Omega \int_0^{E_1} f_{\gamma,k-1}(E) \cdot \left(Tr_i(E) - Tr_j(E)\right) \cdot S_{IP}(E) \cdot dE \quad (12)$$

$$I_{2,k-1} = \theta_z \cdot \Delta\Omega \int_{E_2}^{+\infty} f_{\gamma,k-1}(E) \cdot \left(Tr_i(E) - Tr_j(E)\right) \cdot S_{IP}(E) \cdot dE. \quad (13)$$

The completion of the calculations can be defined by a condition for the difference between the previous and new iterative distribution functions, for example, $\delta < 0.05$ (5%), where:

$$\delta \equiv \max(\delta_l) \text{ where } \delta_l = \left|1 - \frac{f_{\gamma,k-1}(E_l)}{f_{\gamma,k}(E_l)}\right| \quad (14)$$

Here, $E_l = \frac{E_1+E_2}{2}$ represents the average energy in the energy window from E_1 to E_2 for the l-th Ross filter pair used. The calculations can be automated using a Python code.

Using multi-IP-filters for spectra evaluation

Furthermore, it is possible to obtain additional data points in the spectrum (for high-energy X-rays) from the measurements of the X-ray signal by the stack of five IPs (Fig. 5a). For this purpose, the Differential Averaged Transmission (DAT) method[8] was used. This method is based on the maximum signals measured on the IP series (M_1, M_2, M_3, M_4, M_5):

$$M_i = \theta_z \int f_\gamma(E) \cdot \Delta\Omega \cdot Tr_i(E) \cdot S_{IP}(E) \cdot dE. \quad (15)$$

Then, the difference between the signals in two adjacent IPs is given by:

$$M_i - M_j = \theta_z \int f_\gamma(E) \cdot \Delta\Omega \cdot \left(Tr_i(E) - Tr_j(E)\right) \cdot S_{IP}(E) \cdot dE. \quad (16)$$

Here, the product of the transmission difference and IP sensitivity $\left(Tr_i(E) - Tr_j(E)\right) \cdot S_{IP}(E)$ depends on the photon energy (Fig. 3S).

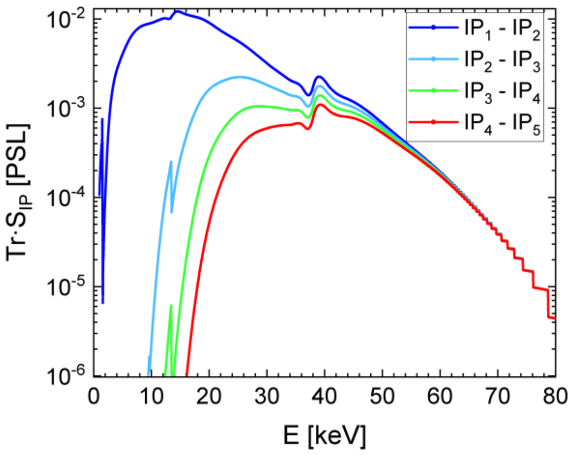

Fig. 3S. Dependence of the product of the transmission difference and IP sensitivity on photon energy for the IP pairs in the consecutive IPs.

For the calculations, the transmission data from the source[60]: https://henke.lbl.gov/optical_constants/ were used for photon energies ranging from 0.5 to 30 keV. For higher energies, the transmission is calculated using the data of the mass attenuation coefficient (μ/ρ) and the density of the filter material ρ from the source[61]: https://physics.nist.gov/PhysRefData/XrayMassCoef/tab3.html, according to the formula:

$$Tr = exp\left(-\frac{\mu}{\rho}\rho d\right), \quad (17)$$

where d is the thickness of the filter. If the filter consists of multiple n layers, the formula is given by:

$$Tr = \prod_{k=1}^{n}(Tr_k) = exp\left(-\sum_{k=1}^{n}\left(\frac{\mu_k}{\rho_k}\rho_k d_k\right)\right). \quad (18)$$

Here, k is the index of a filter layer. The sensitivity $S_{IP}(E)$ of BAS-IP MS was taken from[56,62].

From equation (16), an approximation for the measurements in IP_1 and IP_2 can be derived:

$$M_1 - M_2 \cong f_\gamma\big|_{E_{12}} \cdot \theta_z \cdot \Delta\Omega \int_{E_1}^{E_2}(Tr_1(E) - Tr_2(E)) \cdot S_{IP}(E) \cdot dE \quad (19)$$

with the restrictions E_1 and E_2, which are half of the maximum value of $(Tr_1(E) - Tr_2(E)) \cdot S_{IP}(E)$.

Therefore, $f_\gamma|_{E_{12}}$ can be calculated as:

$$f_\gamma|_{E_{12}} \cong \frac{M_1 - M_2}{\theta_z \cdot \Delta\Omega \int_{E_1}^{E_2} (Tr_1(E) - Tr_2(E)) \cdot S_{IP}(E) \cdot dE}. \quad (20)$$

and for IP_2 and IP_3:

$$f_\gamma|_{E_{23}} \cong \frac{M_2 - M_3}{\theta_z \cdot \Delta\Omega \int_{E_2^*}^{E_3} (Tr_2(E) - Tr_3(E)) \cdot S_{IP}(E) \cdot dE} \quad (21)$$

The calculation according to equations (20) and (21) can be performed using a Python code. Thus, the distribution function f_γ is determined by $f_\gamma|_{E_{12}}$, $f_\gamma|_{E_{23}}$ etc.

The error in energy corresponds to the energy window where the value of the product $(Tr_j(E) - Tr_i(E)) \cdot S_{IP}(E)$ for the ij-IP-pair is ≥50% of the maximum value (see Fig. 3S). The systematic error of 20–30% for the number of photons/keV/sr can be roughly estimated by contribution of the signal outside the energy window. The statistical error reaches 10 to 20% as a result of signal noise on the IPs.

References

60. Henke, B.L. *et al.* X-ray interactions: photoabsorption, scattering, transmission, and reflection at E = 50-30000 eV, Z=1-92. *Atomic Data and Nuclear Data Tables.* **54**(**2**), 181-342. https://henke.lbl.gov/optical_constants/ (1993).

61. Hubbell, J.H. and Seltzer, S.M. X-Ray Mass Attenuation Coefficients (NIST Standard Reference Database 126). https://www.nist.gov/pml/x-ray-mass-attenuation-coefficients (1996).

62. Boutoux, G. *et al.* Validation of modelled imaging plates sensitivity to 1-100 keV x-rays and spatial resolution characterisation for diagnostics for the “PETawatt Aquitaine Laser”. *Rev. Sci. Instrum.* **87**, 043108. http://dx.doi.org/10.1063/1.4944863 (2016).
